# Safety and Comfort of an Innovative Drug Delivery Device in Healthy Subjects

**DOI:** 10.1167/tvst.9.13.35

**Published:** 2020-12-18

**Authors:** Christian J. F. Bertens, Suryan L. Dunker, Aylvin J. A. A. Dias, Frank J. H. M. van den Biggelaar, Rudy M. M. A. Nuijts, Marlies Gijs

**Affiliations:** 1 Chemelot Institute for Science and Technology (InSciTe), GS Maastricht, The Netherlands; 2 University Eye Clinic Maastricht, Maastricht University Medical Center+ (MUMC+), HX Maastricht, The Netherlands; 3 Maastricht University, School for Mental Health and Neuroscience, University Eye Clinic Maastricht, ER Maastricht, The Netherlands; 4 Eyegle bv. Gerbergaplantsoen 11, Maastricht, The Netherlands

**Keywords:** ocular coil, comfort and safety study, placebo drug delivery device, clinical study, ophthalmology

## Abstract

**Purpose:**

The aim of this study was to investigate safety and comfort of two versions of a placebo-microsphere filled ocular coil (straight and curved) in healthy subjects.

**Methods:**

The study was a single-center intervention study. One ocular coil was placed in the inferior conjunctival fornix for the intended duration of 28 days. Forty-two healthy adult subjects were included. At baseline, 30 minutes, 8 hours, 24 hours, 48 hours, 7 days, 14 days, 21 days, and 28 days after insertion, examinations were performed, including slit lamp evaluation to score ocular redness, intraocular pressure measurement, visual acuity, tear secretion test, and questionnaires.

**Results:**

The straight and curved ocular coils had a median retention time of 5 days and 12 days, respectively. After 48 hours, 57% and 81% subjects retained the straight and curved ocular coil, respectively. Four (19%) subjects with the straight coil and six (29%) with the curved coil completed the entire study period. Minor changes in ocular hyperemia were observed in both groups. On day 7, the straight coil was more comfortable than the curved coil with a visual analogue scale (VAS) score of 77 ± 21 compared to 94 ± 11 (*P* = 0.028), respectively. No other ocular adverse events were observed.

**Conclusions:**

Comfort and safety of the straight and curved ocular coil are high. Because the retention time is too short for long-term sustained drug release, the use in the perioperative or immediate postoperative period could prove to be more valuable.

**Translational Relevance:**

The ocular coil is a noninvasive, comfortable and safe short-term drug delivery device.

## Introduction

**Figure 1.**
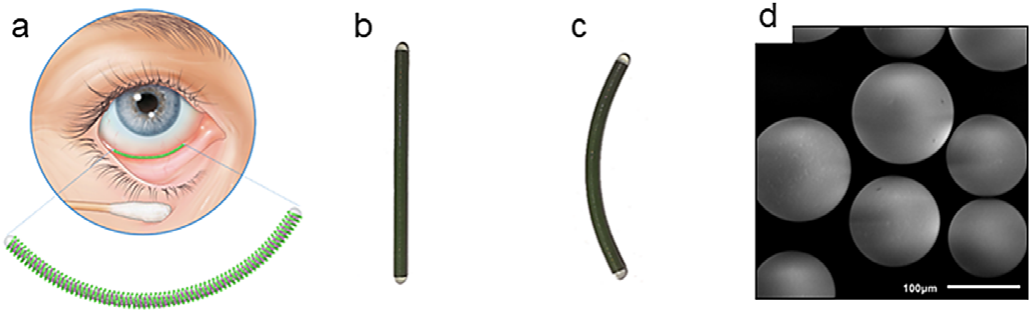
(**a**) Location of the ocular coil in the inferior conjunctival fornix. (**b**) Photograph of a straight ocular coil and (**c**) a curved ocular coil. (**d**) Scanning electron microscopic (SEM) photograph of the microsphere filling of the ocular coil (SEI, 1 kV, 220 × magnification).

Cataract surgery is one of the most performed surgeries in Western society.^1^ To prevent postoperative complications, patients are treated with anti-inflammatory drugs for a period up to 28 days.^1^^–^^3^ Postoperative drugs are mainly administered topical, via eye drops^4^ because of their low costs and ease of use. However, the use of eye drops has several drawbacks. Besides systemic side effects^5^ and local toxicity due to preservatives,^6^^,^^7^ the main disadvantages of eye drops include low bioavailability^8^^–^^10^ and poor patient compliance.^11^^–^^13^ In order to address these problems, our group developed an ocular drug delivery device, the ocular coil. It is designed to rest in the inferior conjunctival fornix (Fig. 1a) in a noninvasive way and can be worn for a specific period of time. The benefits of a noninvasive drug delivery system are that it removes the burden of daily administrating topical drugs and, thereby, increases patient compliance.^14^^–^^17^ The ocular coil is made from a coiled and coated stainless steel wire that is closed at both ends with a dome-shaped UV-curable acrylate urethane cap (Fig. 1b). The inner lumen of the ocular coil can be filled with a drug-eluting matrix for slow and sustained drug release.^18^ For example, we developed ketorolac entrapped poly(methyl methacrylate) (PMMA) microspheres and inserted those into the inner lumen of the ocular coil. Release of ketorolac from the ocular coil occurred via diffusion from the microspheres. In an in vitro lacrimal system, a high dose of ketorolac was released (approximately 50% of the total loading) during the first 3 days, followed by sustained release until day 28.^18^ Pilot studies showed that the ocular coil loaded with an atropine-releasing coating is able to achieve mydriasis,^14^ and that the ocular coil is safe and comfortable to wear for 2 hours.^17^ The aim of the current clinical trial was to evaluate the safety and comfort of a straight and a curved ocular coil for an intended period of 28 days. In this study, we used an ocular coil that was filled with placebo-microspheres (Fig. 1d). Two versions of the ocular coil were evaluated. Initially, a straight ocular coil was designed to bend during wearing (see Fig. 1b), followed by a curved ocular coil that was produced with an inherent curvature according to the outer circumference of the eye (see Fig. 1c).

## Materials and Methods

### Study Design

The study was designed as a unilateral randomized single-center intervention study. The study protocol was approved by the local ethics committee and the national authorities (number: NL57050.068.16/METC161042). The study procedures were performed in accordance with the tenets of the Declaration of Helsinki. The study was registered with the US National Institutes of Health Clinical Trials (ClinicalTrials.gov Identifier: NCT03488017).

### Study Population

Initially, the study was designed as a proof-of-concept study for the straight ocular coil in 40 subjects. However, after observing high occurrence of loss of the ocular straight coil in 21 subjects, inclusion was stopped and the ocular coil was redesigned to a curved ocular coil. After obtaining additional ethical approval, another 21 subjects were included to evaluate the curved ocular coil.

Subjects were included at the University Eye Clinic Maastricht, Maastricht, The Netherlands. From June 2018 until July 2019, 42 healthy adult subjects (between the age of 18 and 75 years) were included for the study with the ocular coil. All subjects gave written informed consent before inclusion. One eye per subject was included and one ocular coil was administered per eye. Exclusion criteria were any history of eye disease, allergies and hypersensitivity of the eye, current use of eye drops, contact lens use, inability to speak or write Dutch, Asian ethnicity (due extra subcutaneous fat in the eyelids), pregnant or breastfeeding women, or women with the intention of becoming pregnant during the study.

### Study Procedures

Before subjects were invited for a screening visit, the inclusion and exclusion criteria were checked. Subjects eligible for participation signed informed consent and underwent a screening session. The screening included an extensive ophthalmologic examination, slit lamp evaluation and photography, intraocular pressure (IOP) measurement (Icare-PRO, Vantaa, Finland), corneal topography (Pentacam HR; Oculus, Irvine, CA), Schirmer's tear production test II (TEARstrips; Contacare Ophthalmics & Diagnostics, Gujarat, India), and visual acuity (best-corrected and uncorrected) using the Early Treatment Diabetic Retinopathy Study (ETDRS) chart.^19^ Moreover, subjects were asked to complete the National Eye Institute Visual Function Questionnaire-25 (VFQ-25 version 2000)^20^ with six detailed questions about ocular discomfort (Supplementary Table S1).

At all visits, slit lamp evaluation (conjunctival and limbal hyperemia, corneal neovascularization, and edema) was performed using a Haag-Streit BX900 slit lamp bio-microscope (Haag Streit AG, Bern, Switzerland) to score according to the Efron grading scale (ranging from 0 = normal to 4 = severe).^21^ Furthermore, conjunctival and corneal punctate staining was scored according to Bron et al.,^22^ and anterior chamber cells and flare were scored using the Standardization of Uveitis Nomenclature (SUN) classification.^23^ Corneas were stained to visualize epithelial damage using fluorescein (Bausch & Lomb, Rochester, NY). Additionally, subjects were asked to complete a customized questionnaire (Supplementary Table S2).^16^ Comfort of the ocular coil was scored using the visual analogue scale (VAS, 0–100; see Supplementary Table S2).

**Figure 2.**
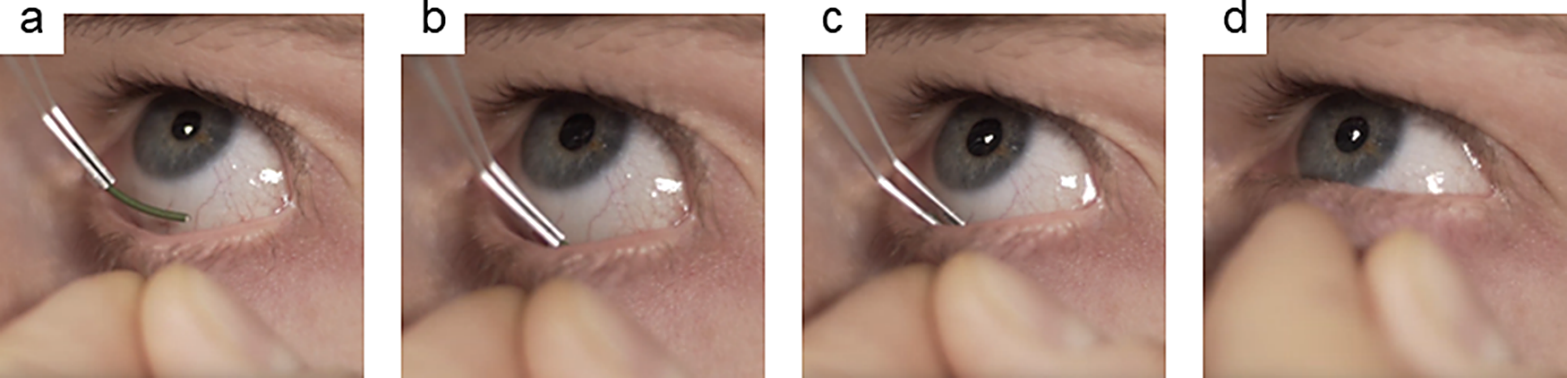
Insertion of the ocular coil. A pocket is made using index finger and thumb (**a**) and the ocular coil is diagonally inserted into the fornix (**b**). The ocular coil was gently released into the fornix (**c**), after insertion, the lower eyelid is released (**d**) and, after a blink, the ocular coil lies in place.

Using a computer algorithm, one eye of each subjects was randomly selected for insertion of the ocular coil. A trained physician inserted the ocular coil in the inferior conjunctival fornix using a Malosa Medical lens folding forceps triangular (#1131; Malosa Limited, Elland, UK) after topical sedation with Oxybuprocaine hydrochloride (MINIMS; Bausch & Lomb Pharma, Brussels, Belgium). The lower eyelid was retracted using the thumb and index finger and the ocular coil was gently placed into the fornix (Fig. 2).

After insertion of the ocular coil, eyes of subjects were evaluated at 30 minutes, 8 hours, 24 hours, 48 hours, 7 days, 14 days, 21 days, and 28 days, and after the ocular coil was removed. When intermediate loss of the ocular coil occurred (and was noticed by the subject), the subject was invited for a close-out visit. When loss of the ocular coil was noticed during one of the follow-up visits (unnoticed by the subject), data from the previous visit was used as the last day that the ocular coil was worn. After inclusion of the 13th subject, a medical eye shield (Dispo Medical BV, Hattemerbroek, The Netherlands) was introduced to prevent unintentional eye rubbing and dislodging of the ocular coil during sleep.

### Outcome Parameters

The primary outcome parameters of the study were conjunctival and limbal hyperemia, corneal defects, and ocular inflammation as determinants of the safety of the ocular oil. Secondary objectives were ocular coil retention time, subject comfort (tolerance) and pain, and incidence of adverse effects and complications (punctate keratitis, conjunctivitis, conjunctival or corneal erosion, and corneal ulceration).

### Statistical Analysis

In this study, two shapes of the ocular coil were tested. Originally, 40 subjects were planned to evaluate the straight ocular coil. However, due to low retention, a redesign of the shape of the ocular coil was needed. This resulted in a lower number of subjects and insufficient statistical power to evaluate safety parameters of the ocular coil.

Difference in age between the study populations for the straight and curved ocular coil was tested using an unpaired *t*-test. Difference in gender and study eye between the two study arms was tested with the χ^2^ test. Retention time of the straight and curved ocular coils was compared using the Mantel-Cox log rank test. Mean and median of the retention time were tested using an unpaired *t*-test and a Mann Whitney rank sum test, respectively.

Due to the high number of missing data (due to variable loss of the coil), three complete case analyses were performed (i.e. for subjects who had a retention time up to 48 hours, up to 7 days, and for subjects who completed the entire study of 28 days).

Comparison of comfort of both ocular coils was done using multiple *t*-tests with a Bonferroni correction for multiple testing.

Tear migration length was compared using a paired *t*-test.

## Results

### Study Population

**Figure 3.**
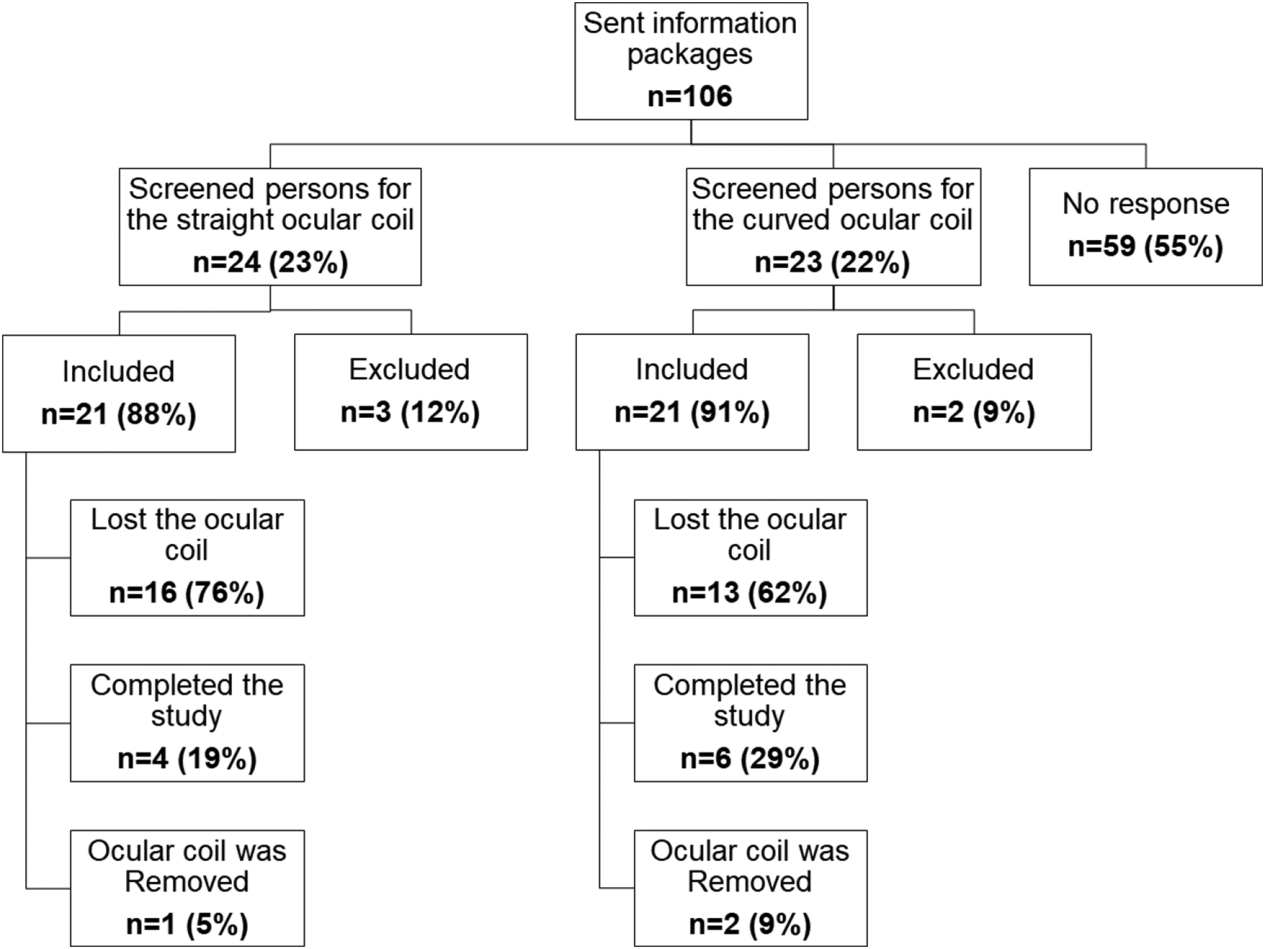
Flow diagram showing the number of subjects who were screened, included, randomized, and analyzed for both studies.

Figure 3 shows a flow diagram of the number of subjects who were approached, screened, included, randomized, and analyzed in the study. In total, 106 information packages were sent to persons that showed interest to participate. In total, 47 (45%) of the interested persons were invited for screening. During screening, 5 subjects (21%) were found not eligible for participation due to their ocular condition, and 42 healthy subjects were included in the study.

Demographics of the subjects are shown in Table 1. In the straight ocular coil arm, 12 subjects (57%) and 9 subjects (43%) received the ocular coil in their right and left eyes, respectively. In the curved ocular coil arm of the study, 10 subjects (48%) received an ocular coil in the right eye and 11 (52%) received an ocular coil in the left eye. The percentage of female subjects’ study who received the curved versus the straight ocular coil was 67% and 52%, respectively.

**Table 1.** Subject Characteristics for Both Versions of the Ocular Coil

Parameter	Straight Coil	Curved Coil	*P* Value
Mean age ± SD, y	53 ± 19	55 ± 19	0.83
Range age, min–max, y	22–74	21–74	N.A.
Gender ratio, male (%)/female	♂ 10 (48%)/♀ 11 (52%)	♂ 7 (33%)/♀ 14 (67%)	0.35
Study eye OD (%)/OS	12 (57%)/9 (43%)	10 (48%)/11 (52%)	0.54

Difference in age is tested using unpaired students t-test, gender difference and study eye is tested using χ^2^ test.

N.A., not applicable.

### Retention

**Figure 4.**
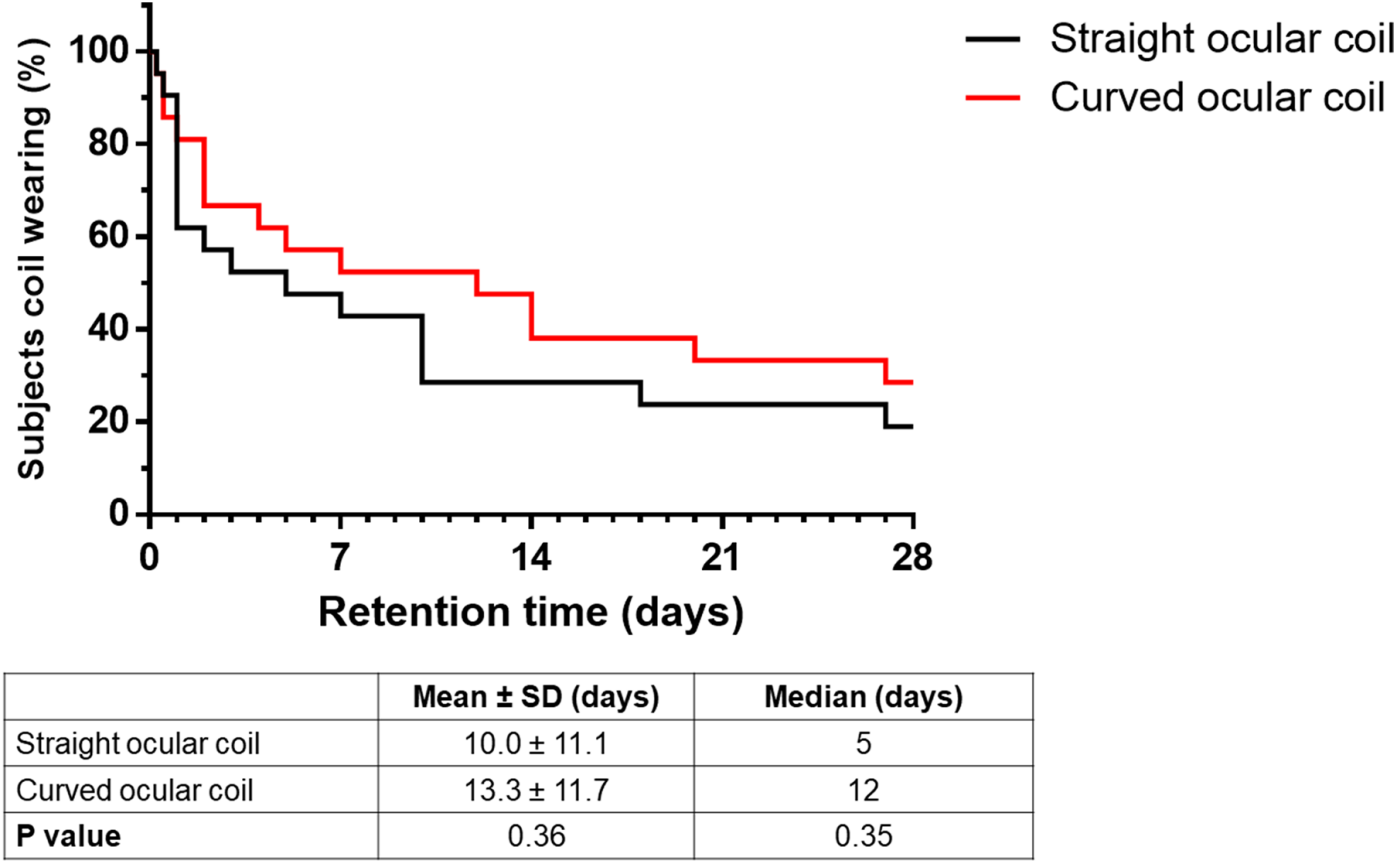
Retention of the straight and curved ocular coil during the study period of 28 days. *P* = 0.38 using the Mantel-Cox test. Testing difference between the means using Students *t*-test *P* = 0.36 and difference between median using Mann Whitney rank test *P* = 0.35.

Retention is defined as the period of time a subject was wearing the ocular coil. Retention of the straight and curved ocular coil is depicted in Figure 4. For the straight ocular coil, 2 of 21 subjects lost the ocular coil within 1 day. After 48 hours and 1 week, 12 (57%) and 10 (47%) of 21 subjects were still wearing the straight ocular coil, respectively. Four (19%) subjects succeeded to wear the straight ocular coil for the full study period of 28 days.

For the curved ocular coil, the retention is also plotted in Figure 4. Three subjects lost the ocular coil within 1 day. After 48 hours, 17 (81%) subjects were wearing the curved ocular coil, after 1 week, 12 (57%) subjects were still wearing the ocular coil. Six (29%) subjects have worn the curved ocular coil for the full study period of 28 days.

No statistical difference (*P* = 0.38) in retention time between the straight and the curved ocular coil was observed. For the curved coil as compared to the straight coil, mean retention time slightly increased from 10 ± 11 days to 13 ± 12 days (*P* = 0.36), and median retention time increased from 5 days to 12 days (*P* = 0.35), respectively (see Fig. 4).

Reasons for loss of the curved and straight ocular coils are listed in Table 2. Eye rubbing was the major cause of loss of the ocular coil in the straight ocular coil group, whereas a majority of subjects in the curved ocular coil group where not aware of loss. One subject removed the ocular coil from the eye after it protruded nasally.

**Table 2.** Reasons for Loss of the Ocular Coil

Reasons for Loss of the Ocular Coil	Straight Coil (*n* = 17/21)	Curved Coil (*n* = 15/21)
Eye rubbing/manipulating the eye	7	1
During sleep (without eye shield)	3	N.A.
During sleep (with eye shield)	0	3
Changing clothes	1	1
Checking whether the ocular coil was still in the fornix	2	-
Removed the coil because of nasal protrusion	1	-
Unknown reason	1	9
Removed upon request	2	1

N.A., not applicable.

In three cases, the ocular coil was removed upon request. In the first case, the ocular coil was removed on the day of insertion because the subject complained about pain after getting a twig (from a tree) in his/her eye. Ocular examination revealed a corneal erosion (Supplementary Figure S1). In the second case, the ocular coil was removed after 14 days due to foreign body sensations, and in a third case the ocular coil was removed because it migrated to the upper eyelid, causing irritation (Supplementary Figure S2a).

### Safety

**Figure 5.**
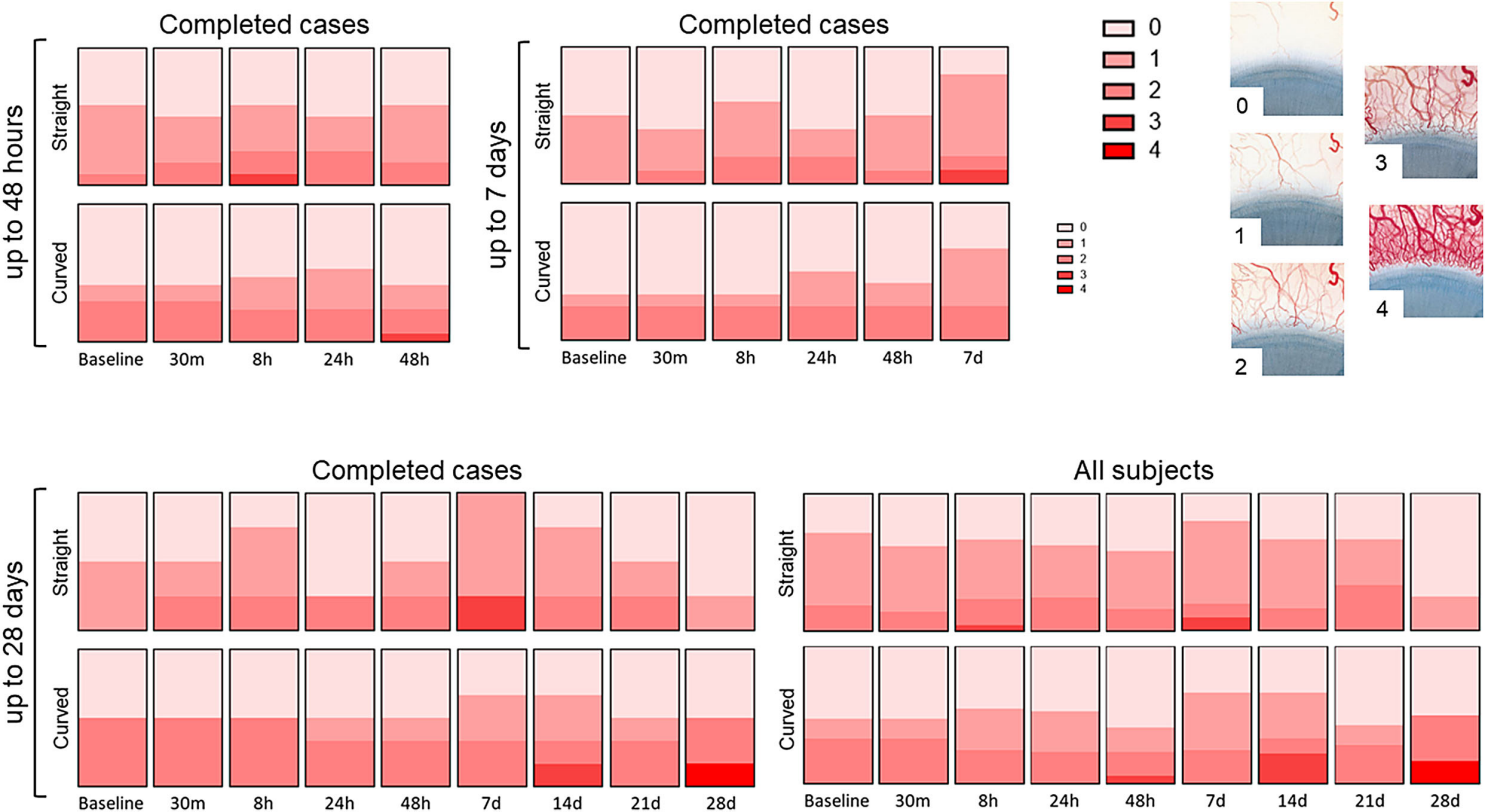
Conjunctival hyperemia according to the Efron grading scale 21. Complete case analysis at 48 hours (n_straight_ = 12, n_curved_ = 17), 7 days (n_straight_ = 10, n_curved_ = 12), and 28 days (n_straight_ = 4, n_curved_ = 6), and the analysis of all subjects at 28 days (n_straight_baseline_ = 21, n_curved_baseline_ = 21) for the straight and curved ocular coils.

Conjunctival hyperemia is plotted in Figure 5. The mean hyperemia score for subjects wearing the straight ocular coil and the curved ocular coil for the first 48 hours was 0.75 ± 0.75 and 0.71 ± 0.99, for the 7 day period was 0.68 ± 0.75 and 0.68 ± 0.85, and for the 28 day period was 0.78 ± 0.83 and 1.00 ± 1.05, respectively. For the first 48 hours, conjunctival hyperemia was similar for both ocular coils. At 7 days, conjunctival hyperemia slightly lowered for both ocular coils, however, hyperemia of the curved ocular coil seems to show less fluctuations compared to the straight ocular coil. One subject wearing a straight ocular coil had a conjunctival hyperemia score of “3” (moderate) at day 7 for unknown reasons that did not lead to other complaints. Two other subjects wearing a curved ocular coil presented with increased conjunctival hyperemia on day 14 and day 28, respectively. The latter was related to a hyposphagma due to eye rubbing (Supplementary Figure S3).

**Figure 6.**
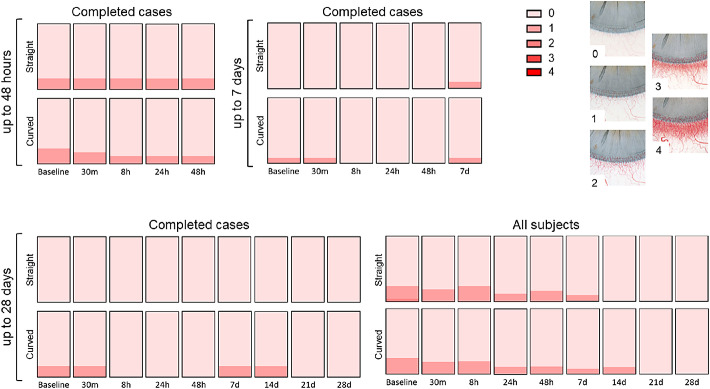
Limbal hyperemia according to the Efron grading scale 21. Completed cases for 48 hours (n_straight_ = 12, n_curved_ = 17), 7 days (n_straight_ = 10, n_curved_ = 12), 28 days (n_straight_ = 4, n_curved_ = 6), and the analysis of all subjects at 28 days (n_straight_baseline_ = 21, n_curved_baseline_ = 21) for both the straight and curved ocular coils.

**Figure 7.**
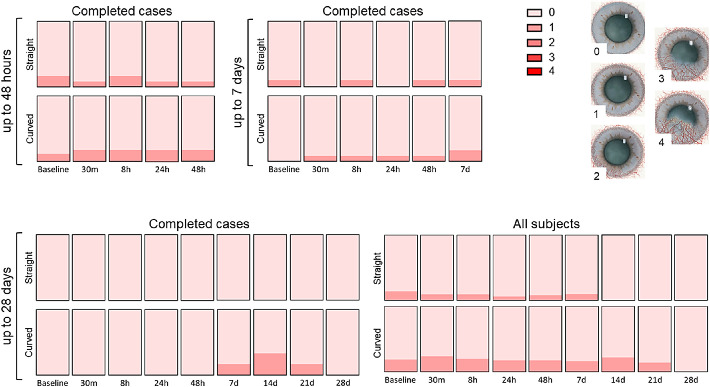
Corneal neovascularization according to the Efron grading scale 21. Completed cases for 48 hours (n_straight_ = 12, n_curved_ = 17), 7 days (n_straight_ = 10, n_curved_ = 12), 28 days (n_straight_ = 4, n_curved_ = 6), and the analysis of all subjects at 28 days (n_straight_baseline_ = 21, n_curved_baseline_ = 21) for both the straight and curved ocular coils.

Only minor changes were observed when scoring limbal hyperemia (Fig. 6). This also applied to corneal neovascularization (Fig. 7). A slight increase in neovascularization was observed in the curved ocular coil group but disappeared at day 28.

No signs of anterior chamber inflammation were noticed with a maximum of one cell observed (SUN guidelines 23) in the anterior chamber, and no presence of flare in any subject during the study (data not shown). Visual acuity, IOP, and corneal topography of all subjects did not differ at any visit compared to baseline (data not shown).

### Comfort

**Figure 8.**
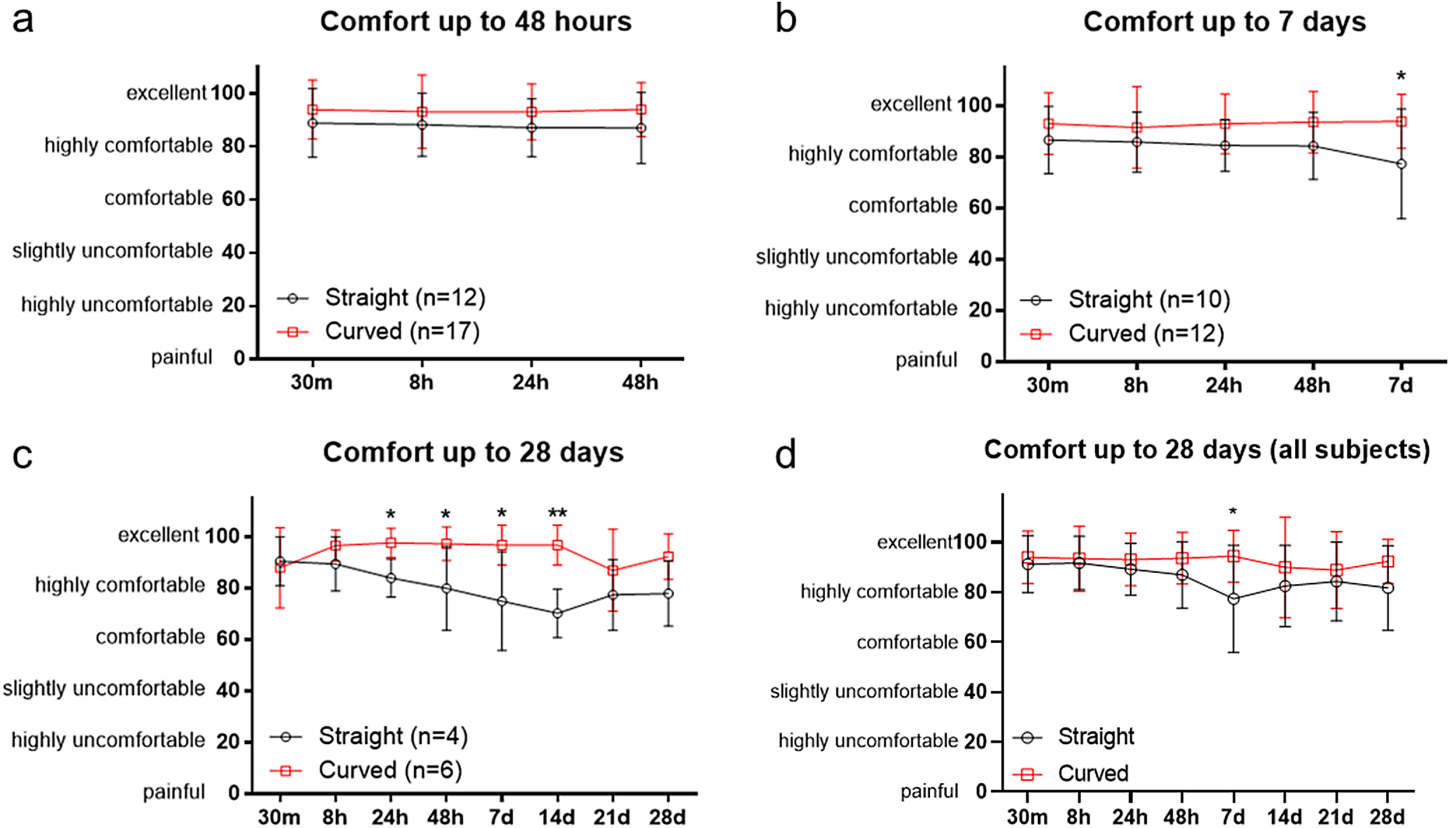
Comfort of the ocular coils as a complete case analysis up to 48 hours (**a**) 7 days (**b**) and 28 days (**c**). Data from all subjects up to 28 days (**d**). Data is shown as mean ± SD, * *P* < 0.05, ** *P* < 0.01 tested using multiple *t*-tests with Bonferroni correction for multiple comparisons.

Comfort was scored at each follow-up visit through a questionnaire and a VAS score. Figure 8 shows comfort of both ocular coils as complete case analysis for the first 48 hours (see Fig. 8a), up to day 7 (see Fig. 8b), and day 28 (see Fig. 8c), whereas Figure 8d shows comfort of all subjects. Overall, both ocular coils were found comfortable to wear during the first 48 hours (see Fig. 8a). Although both coils were considered highly comfortable to excellent, the curved ocular coil was more comfortable at day 7 compared to the straight ocular coil (VAS of 77 ± 21 compared to 94 ± 11, *P* = 0.028, respectively; see Fig. 8b). Furthermore, the curved ocular coil showed less fluctuations in comfort between 30 minutes and 7 days.

For subjects that completed the study, the curved ocular coil was more comfortable after 24 hours (VAS score of 84 ± 7 vs. 98 ± 6; *P* = 0.011), 48 hours (VAS score of 80 ± 16 vs. 97 ± 7, *P* = 0.044), 7 days (VAS score of 75 ± 19 vs. 97 ± 8; *P* = 0.034), and 14 days (VAS score of 78 ± 17 vs. 97 ± 8, *P* = 0.001; see Fig. 8c) as compared to the straight coil. The curved coil also provided less fluctuation in comfort over a period of 28 days compared to the straight ocular coil. No statistical difference in comfort between 30 minutes and 28 days was observed. Comparing all subjects, significant difference in comfort between the straight and curved ocular coil is only found on day 7 (VAS score of 77 ± 21 vs. 94 ± 10, *P* = 0.0019; see Fig. 8d).

**Figure 9.**
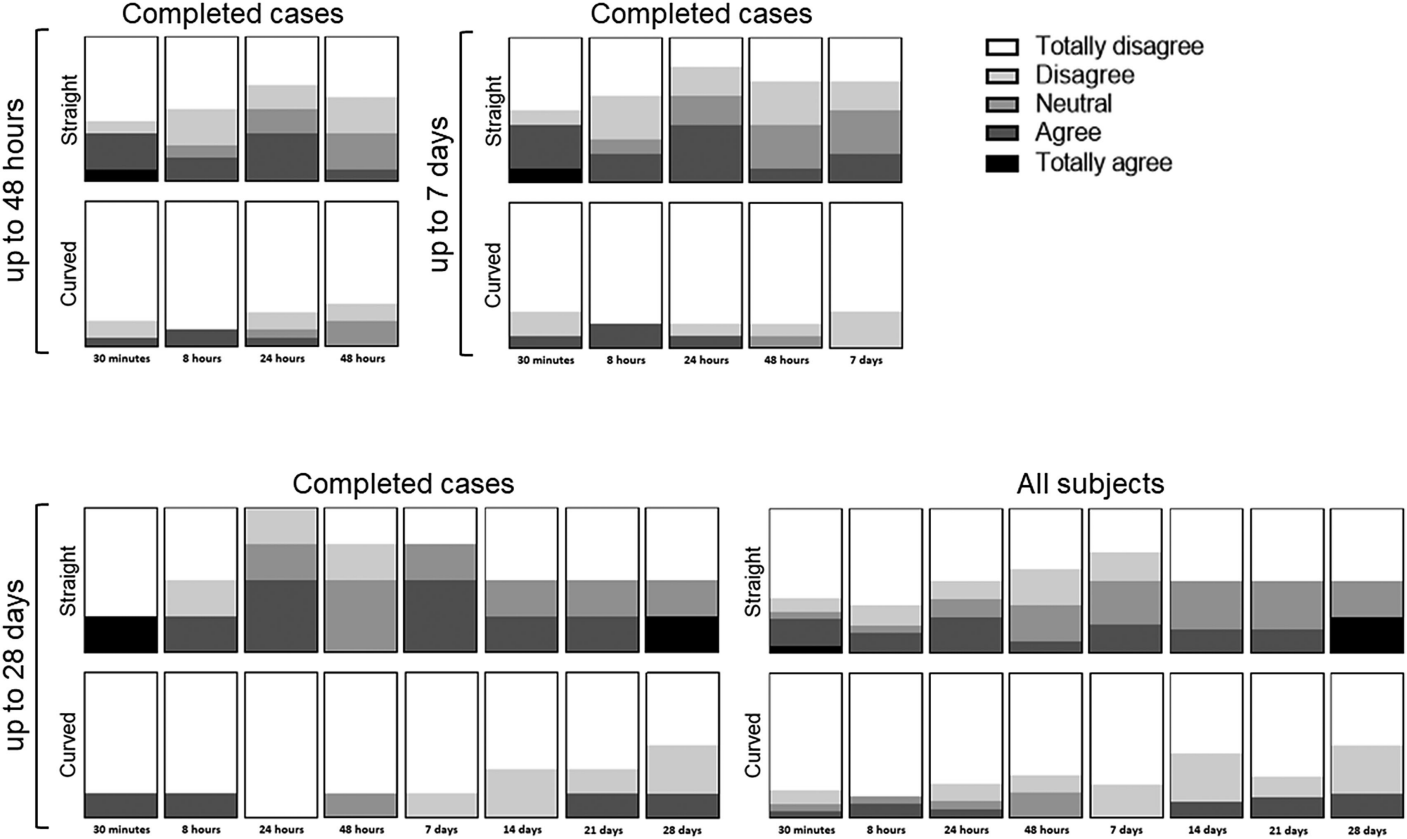
Questionnaire “I feel the presence of the ocular coil in my eye.” Completed cases for 48 hours (n_straight_ = 12, n_curved_ = 17), 7 days (n_straight_ = 10, n_curved_ = 12), 28 days (n_straight_ = 4, n_curved_ = 6), and the analysis of all subjects at 28 days (n_straight_baseline_ = 21, n_curved_baseline_ = 21) for both the straight and curved ocular coils.

**Figure 10.**
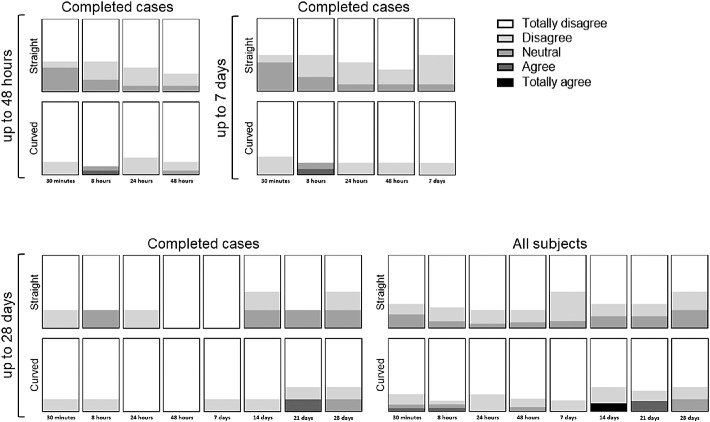
Questionnaire “Presence of the ocular coil in my eye is uncomfortable.” Completed cases for 48 hours (n_straight_ = 12, n_curved_ = 17), 7 days (n_straight_ = 10, n_curved_ = 12), 28 days (n_straight_ = 4, n_curved_ = 6), and the analysis of all subjects at 28 days (n_straight_baseline_ = 21, n_curved_baseline_ = 21) for both the straight and curved ocular coils.

During the follow-up moments, the subjects were asked several questions (see Supplementary Table S2), such as whether they feel the ocular coil (Fig. 9) and whether it is uncomfortable to have the ocular coil in their fornix (Fig. 10). Overall, more persons noted the ocular coil in their eye in the straight ocular coil group compared to the curved ocular coil group. The presence of the straight ocular coil was considered slightly more uncomfortable than the curved ocular coil. At day 14, one subject found the ocular coil uncomfortable to wear, therefore, the ocular coil was removed upon request, due to foreign body sensations.

**Figure 11.**
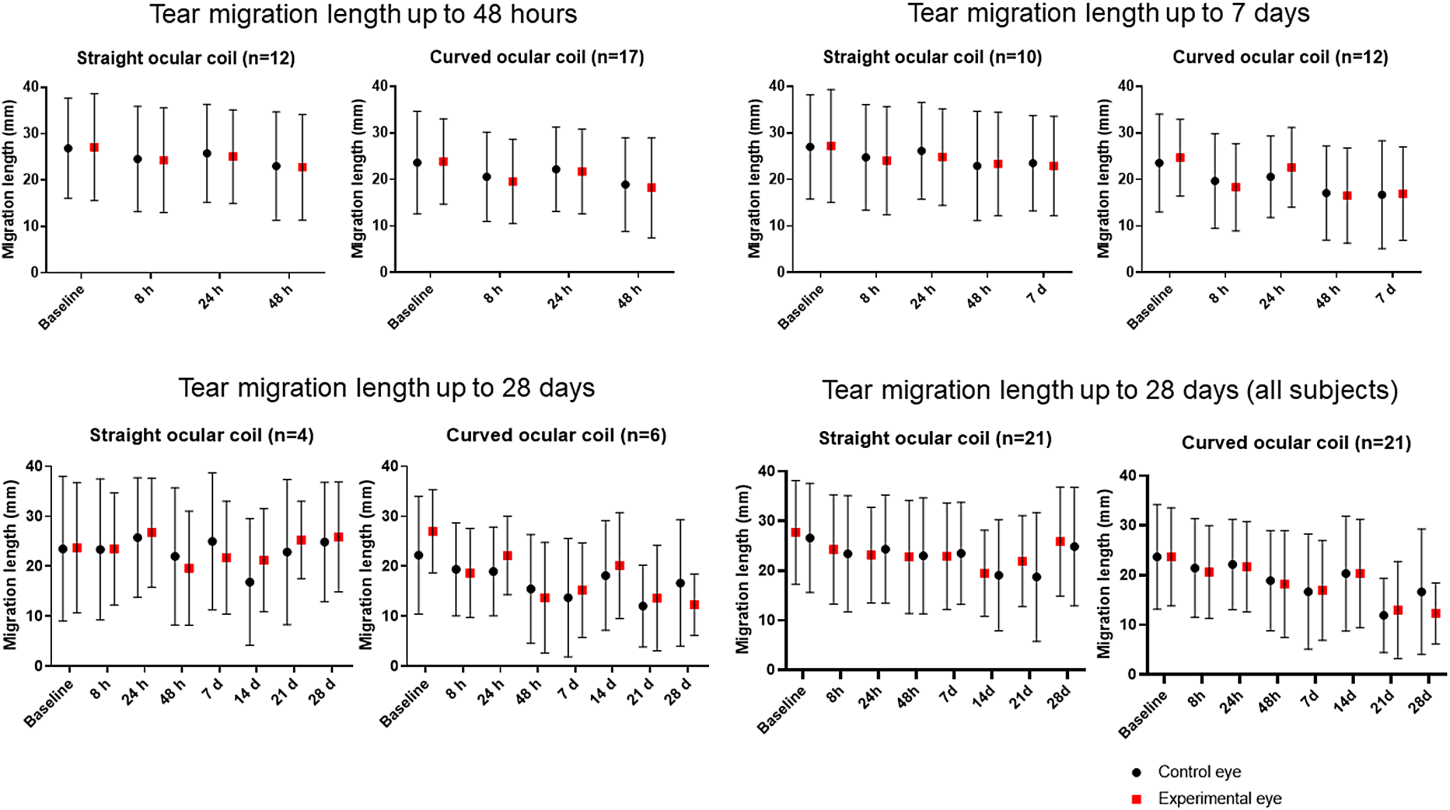
Schirmer's tear production test (II) for the study eye (*red square*) and control eye (*black dot*). Completed cases for 48 hours (n_straight_ = 12, n_curved_ = 17), 7 days (n_straight_ = 10, n_curved_ = 12), 28 days (n_straight_ = 4, n_curved_ = 6), and the analysis of all subjects at 28 days (n_straight_baseline_ = 21, n_curved_baseline_ = 21) for the straight and curved ocular coils.

Subjects were asked whether their eyes teared more frequently while wearing the ocular coil. The majority of subjects did not experience increased tearing. One subject wearing the straight ocular coil went from “sometimes,” to “often,” and one went from “sometimes” to “continuously” after 30 minutes, however, this returned to baseline level at 8 hours. Few subjects wearing the straight ocular coil reported a mild increase in tearing, whereas the curved ocular coil subjects stayed stable compared to baseline (Supplementary Figure S4). Tear production was also objectively assessed using a Schirmer's tear production test (Fig. 11). In contrast to an increased tearing experience of a few subjects, no significant difference between the control eye and study eye was observed using the Schirmer's test. There was no significant change over time in both study arms.

### Adverse Events

All adverse events are shown in Table 3. No serious adverse events were reported during the course of the study. Forty-three percent of the subjects (at both ocular coils) experienced migration of the ocular coil toward the caruncle (see Supplementary Figure S2b). Adverse events included corneal erosion (see Supplementary Figure S1), dislocation of the ocular coil, ocular irritation, transient blurred vision, painful or foreign body sensations, ocular discharge, and headache. Dislocation of the curved ocular coil toward the superior conjunctival fornix was observed in three (14.3%) subjects (see Supplementary Figure S3a). Within these three cases, one dislocated ocular coil was removed whereas two ocular coils were repositioned.

**Table 3.** Adverse Events Association with Wearing the Ocular Coil

	Straight Coil *n* (%)	Curved Coil *n* (%)
**Ocular adverse events**		
Ocular irritation	1 (5%)	–
Corneal erosion	1 (5%)	–
Transient blurred vision	1 (5%)	–
Painful or foreign body sensations	1 (5%)	1 (5%)
Dislocation of the ocular coil toward the caruncle	9 (43%)	9 (43%)
Dislocation of the ocular coil to the superior fornix	–	3 (14%)
Ocular discharge	3 (14%)	1 (5%)
**Systemic adverse events**		
Headache	1 (5%)	–

## Discussion

This study gives a detailed insight into safety and comfort of the ocular coil. Safety and comfort are essential for a new drug delivery device in order to serve as a functional alternative to eye drops and assure high compliance. In a pilot study, 5 healthy subjects wore 1 ocular coil (filled with hydrogel-coated placebo filaments in its inner lumen) for 2 hours. Although the subjects felt the presence of the ocular coil in the conjunctival fornix, the coil was not scored as unpleasant (mean comfort score of 2.2 ± 1.2 on a scale from 1 = very comfortable to 5 = uncomfortable).^17^ In addition, the eye did not show signs of ocular irritation.^17^

In this study, two new versions of the ocular coil (filled with placebo microspheres) were tested. A small number of subjects felt the presence of the ocular coil in the conjunctival fornix. This number increased over time as the subjects became more aware of the straight ocular coil. In contrast, the curved ocular coil was only minimally felt in the fornix. We therefore questioned the subjects whether presence of the ocular coil was uncomfortable and whether the subjects were hindered in their daily tasks by the ocular coil. Presence of the ocular coil was felt but wearing the ocular coil was not considered annoying nor did it hinder the subjects during their daily tasks. Although both ocular coils were considered comfortable, the curved coil provided a more stable comfort score over the full duration of the study.

Safety of the ocular coil is another important factor. To exclude drug-related side effects of a drug delivery device, the ocular coil was tested with placebo microspheres in healthy subjects. One of the main symptoms indicating ocular irritation would be conjunctival hyperemia.^24^ Hyperemia was scored using the Efron's grading scale.^21^ Subtle variations in hyperemia were observed during the study. However, placement of the ocular coil did not result in acute hyperemia, nor was there chronic irritation resulting in an increase in hyperemia after wearing the ocular coil for multiple weeks. On day 28, one of the subjects rubbed his eye, which resulted in a hyposphagma (see Supplementary Figure S3). It is difficult to conclude whether the hyposphagma occurred due to the presence of the ocular coil or only due to eye rubbing. Similarly, it was hard to judge whether the corneal erosion in another subject was due to dislocation of the ocular coil or to a twig from a tree that the subject accidentally got in his eye. In both cases, we cannot rule out that dislocation of the coil contributed to the occurrence of the corneal epithelial defects. The advantageous noninvasive (and mobile) nature of the ocular coil, therefore, also has its drawbacks impeding future clinical applications. The risk of complications due to dislocation could be minimized by increasing further the biocompatibility of the coil (e.g. modify the coating to decrease the friction of the surface), and by optimizing the device's design in order to prevent (sharp) edges and irregular interfaces.

The Efron's grading scale was created to evaluate contact lens related complications, it enabled us to carefully score and track ocular changes related to the ocular coil. Our study showed an average conjunctival hyperemia score of 0.78 ± 0.82 and 1.00 ± 1.04 for subjects wearing the straight ocular coil and the curved ocular coil over a period of 28 days, respectively. These results are comparable to the average conjunctival hyperemia scores that were observed in 2 cohorts testing contact lens materials in 20 healthy adult contact lens wearers (i.e. 0.75 ± 0.19 and 0.94 ± 0.25).^25^ Objective scoring of ocular hyperemia, however, remains difficult. Inter- and intra-observer differences are inevitable, particularly in large multicenter studies.^26^ Therefore, our group is developing an automated computer program for objective redness scoring of slit lamp images.^27^

According to the Efron grading scale, a neovascularization score of 1 was also present in 7 subjects at baseline, a finding clearly not related to the presence of the coil. In these seven subjects, no increase in neovascularization was noted during the study. An increase in vascularization from grade 0 to grade 1 was seen in 5 subjects, remained stable in 7 of these subjects, and disappeared in 6 of the subjects. Neovascularization was not accompanied by other symptoms or complaints. We therefore hypothesize that the variation might be contributed due to differences in subjective grading. To rule out that the changes are not caused by the coil itself but due to variations in grading, an objective neovascularization measurement system could be helpful in avoiding the variations inherent of subjective grading systems.

Retention time of the ocular coil in the eye was lower than expected. We noticed that the majority of the subjects lost the ocular coil when they were manipulating their eye (lids; e.g. rubbing or washing). In some subjects, loss of the ocular coil occurred while sleeping. Introducing an ocular eye shield at night did not improve retention. Redesigning the ocular coil from straight to curved to lower tension on the tissue in the fornix did not increase the average retention time (10.0 ± 11.1 days to 13.3 ± 11.7 days) but improved (although not significantly) the median retention time (from 5 to 12 days). However, for a 48-hour period, a retention time of 81% could be achieved using the curved ocular coil.

Devices with other shapes have similar retention issues. The rod-shaped ocular drug delivery device (Ocufit SR, 25–30 mm length, 1.9 mm diameter) could be retained for 2 weeks in the superior conjunctival fornix in 70% of the cases.^28^ Although these retention times are higher than ours (43% of cases for the straight coil and 48% of cases for the curved coil over a 2-week period), we prefer placement of the device in the inferior conjunctival fornix in order to lower the risk for causing corneal damage following blinking of the upper eyelid. Furthermore, placement of the ocular coil in the inferior fornix appears not to interfere with eye muscle movements.^18^

Another study, performed by Katz et al., tested retention of a dissolvable rod and a dissolvable oval shaped drug delivery device for 24 hours tested for 7 days (a new device every day). They found that a rod-like shape is beneficial over an oval shape. Furthermore, 60% of their drug delivery devices were lost upon, or within 1 hour after arising, when subjects inadvertently rubbed their eyes.^29^ In our study, six subjects lost the ocular coils during sleep (15%).

More recently, the bimatoprost ring (also known as Helios; Allergan, Dublin, Ireland) was developed. This ring is inserted in the superior and inferior fornices around the bulbus. The retention time of the bimatoprost ring was 93% at 12 weeks and 88.5% at 6 months.^30^ However, the retention time in their study was defined as maintenance of the insert without requiring physician re-intervention.^30^ In all cases, patients were aware of dislodgement of the bimatoprost ring.^31^ Therefore, patients were instructed to reinsert the bimatoprost ring themselves, which resulted in a learning curve, increasing retention time (from 88% to 97% in 6 to 7 months).^31^ In contrast, our subjects were instructed not to re-insert the ocular coil after loss. Furthermore, when dislocation of the ocular coil was observed by the investigators, 24% of subjects were not aware of this dislocation. Retention time of small devices for the inferior conjunctival fornix is lower compared to ring-like structures.^30^^,^^31^ This may be a problem with any single-fornix ocular devices. Despite different shapes, all these types of (relatively large) drug delivery devices thus seem to share similar problems with dislocation and loss from the eye.

Given the acceptable retention time of 81% over the 48-hour period, a curved coil may be suitable to use for perioperative application during cataract surgery. Currently, there is growing interest in so-called dropless cataract surgery, where drug-loaded devices can provide adequate medical treatment to prevent postoperative inflammation.^32^^–^^34^ In the United States, Imprimis Pharmaceuticals (San Diego, CA) developed TriMoxi (less drops) and TriMoxiVanc (dropless), two compounded injections that consists of Triamcinolone and Moxifloxacin for perioperative use.^32^^,^^34^ They estimated that as such the use of postoperative drops can be avoided in more than 90% of patients.^35^ However, one must take into account the obstructed vision (a “cloud” or “plume”) during the first days to week postoperatively.^32^

Another perioperative solution developed by Omerios Coorperation (Seattle, WA) is Omidria. Omidria contains phenylephrine (1%) and ketorolac (0.3%) and is used in the irrigation fluid during surgery. Omidria stabilizes mydriasis and reduces postoperative pain.^36^ However, Omidria is not intended as prophylaxis for cystoid macular edema. A third injectable is Dexycu, developed by EyePoint Pharmaceutics (Watertown, MA). Dexycu is a 9% dexamethasone suspension to be injected peri-operatively after insertion of the intraocular lens and reduces postoperative inflammation.^37^

Recently, Ocular Therapeutix (Bedford, MA) brought Dextenza on the market, a 0.7 mg dexamethasone containing punctum plug to prevent postoperative inflammation.^33^ Two prospective multicenter studies observed a reduction in ocular pain and inflammation compared to a placebo device.^38^ Ninety-six percent of patients were satisfied with the use of Dextenza and 88% would want to use the insert again after ocular surgery.^39^ These results demonstrate that there is market potential for noninvasive drug delivery devices.

With a retention time of 81% after 48 hours, the curved ocular coil would be suitable to use in the early postoperative phase after ocular surgery. Further studies are needed to investigate its efficacy and applicability.

## Conclusion

This single-center intervention study provides an overview of the safety and comfort of two versions of the ocular coil. The current study indicates a high comfort profile of both ocular coil designs. Whereas safety of the curved ocular coil seems higher than the straight ocular coil because of the occurred adverse events. Retention time of the ocular coils, however, was lower than expected for the 7-day and 28-day periods, but satisfactory for a 48-hour period. This would make the current design suitable for drug delivery in a burst release mode in the early postoperative phase in surgical procedures that elicit a low to moderate inflammatory response like cataract surgery. This potential application will need further investigation.

## Supplementary Material

Supplement 1

Supplement 2

Supplement 3

Supplement 4

Supplement 5

Supplement 6
