# Supplementary material for: Safety and Comfort of an Innovative Drug Delivery Device in Healthy Subjects
Source: Transl Vis Sci Technol. 2020 Dec 18;9(13):35. doi: 10.1167/tvst.9.13.35 (PMC7757610; doi:10.1167/tvst.9.13.35)
Supplement: Supplement 1 [file tvst-9-13-35_s001.pdf]

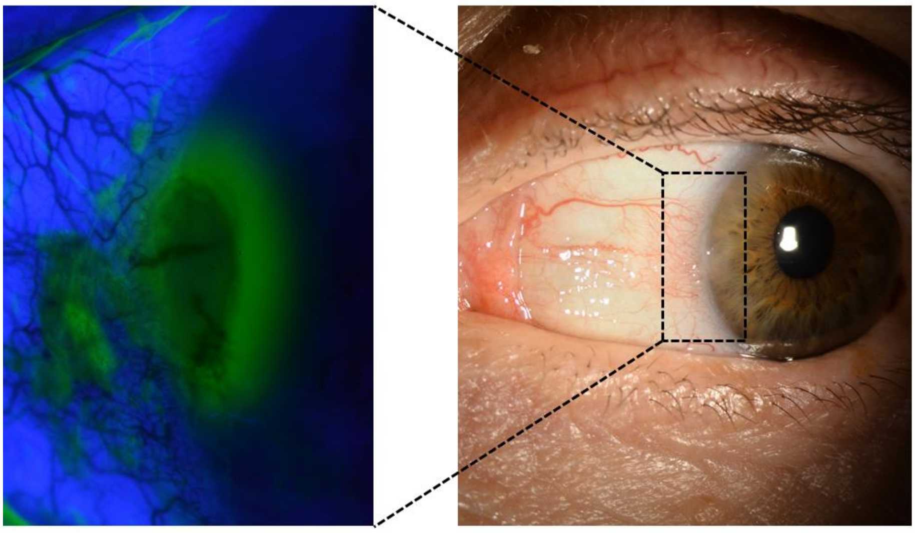

Figure S1. Corneal erosion due to a twig in the eye of a subject (unrelated to the ocular coil). Slit lamp images at 6.3x magnification and insert at 16x magnification stained with fluorescein.
