# Supplementary material for: Safety and Comfort of an Innovative Drug Delivery Device in Healthy Subjects
Source: Transl Vis Sci Technol. 2020 Dec 18;9(13):35. doi: 10.1167/tvst.9.13.35 (PMC7757610; doi:10.1167/tvst.9.13.35)
Supplement: Supplement 2 [file tvst-9-13-35_s002.pdf]

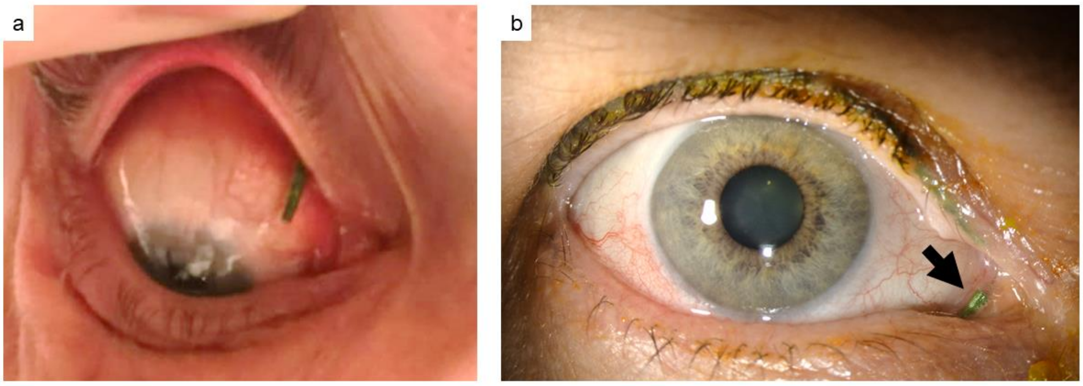

Figure S2. (a) Dislocation of the curved ocular coil to the superior conjunctival fornix, and (b) migration of the ocular coil to the caruncle (indicated with the black arrow). Photo (a) was taken with an iPhone XR.
