# Supplementary figures and images for: Safety and Comfort of an Innovative Drug Delivery Device in Healthy Subjects
Source: Transl Vis Sci Technol. 2020 Dec 18;9(13):35. doi: 10.1167/tvst.9.13.35 (PMC7757610; doi:10.1167/tvst.9.13.35)

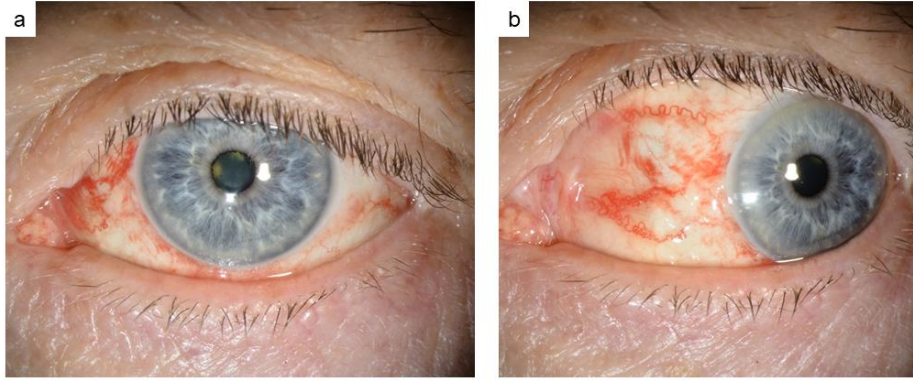

**Figure S3.** Photo of a hyposphagma (a) gazing forward, and (b) left gaze direction.

Supplement: Supplement 3 [file tvst-9-13-35_s003.pdf]
