# Supplementary material for: Safety and Comfort of an Innovative Drug Delivery Device in Healthy Subjects
Source: Transl Vis Sci Technol. 2020 Dec 18;9(13):35. doi: 10.1167/tvst.9.13.35 (PMC7757610; doi:10.1167/tvst.9.13.35)
Supplement: Supplement 4 [file tvst-9-13-35_s004.pdf]

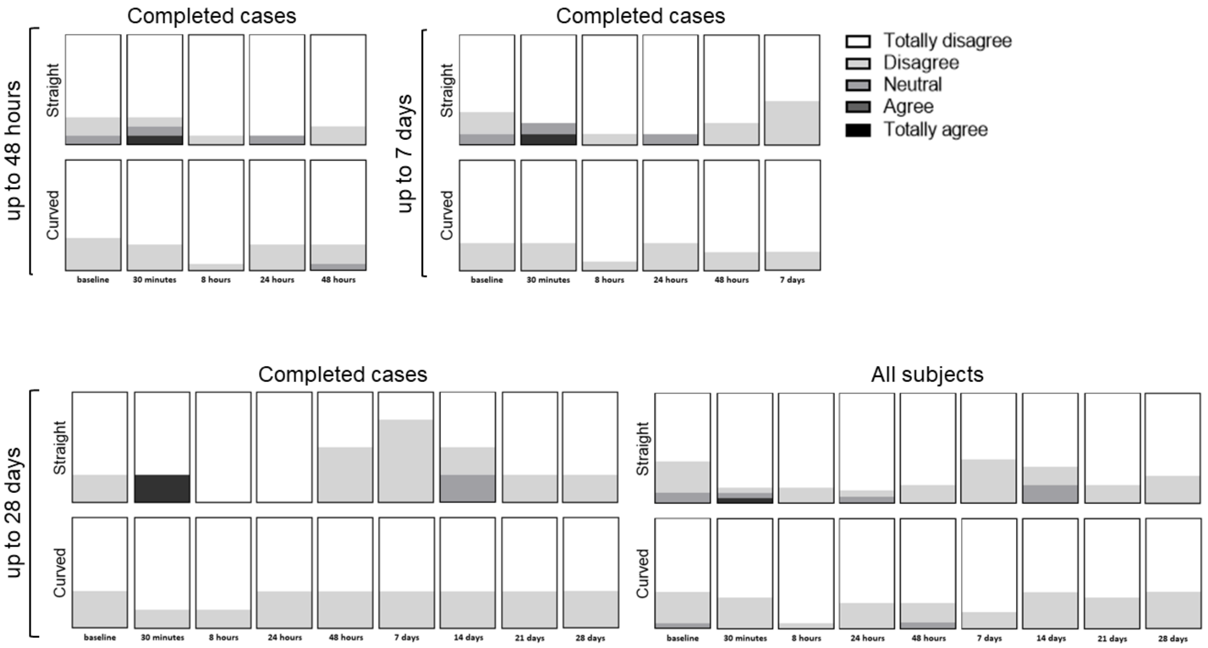

Figure S4. Questionnaire ‘My eye has a high tear production’. Completed cases for 48 hours ($n_{straight}$=12, n_{curved}=17), 7 days ($n_{straight}$=10, n_{curved}=12), 28 days ($n_{straight}$=4, n_{curved}=6), and the analysis of all subjects at 28 days ($n_{straight\_baseline}$=21, $n_{curved\_baseline}$=21) for both the straight and curved ocular coil.
