# Supplementary material for: Safety and Comfort of an Innovative Drug Delivery Device in Healthy Subjects
Source: Transl Vis Sci Technol. 2020 Dec 18;9(13):35. doi: 10.1167/tvst.9.13.35 (PMC7757610; doi:10.1167/tvst.9.13.35)
Supplement: Supplement 5 [file tvst-9-13-35_s005.docx]

**Table S1.** Additional questions VFQ25 version 2001.

| 4a | Was pain or discomfort caused by wearing contact lenses? | - Yes - No |
| --- | --- | --- |
| 4b | Was pain or discomfort caused by ocular surgery? | - Yes - No |
| 4c | Was pain or discomfort caused by use of ocular drugs e.g. eye drops or ointments? | - Yes - No |
| 4d | Was pain or discomfort caused by an allergic reaction e.g. hay fever, cat or dog allergy? | - Yes - No |
| 4e | (if yes on question 4a) Do you wear contact lenses to improve your eyesight? (use ‘no’ for cosmetic reasons) | - Yes - No |
| 4f | Why do you not use contact lenses? | - My eyesight is good therefore I do not need correction. - I prefer glasses - I cannot wear contact lenses due to ocular irritation or discomfort |

*Questions were inserted at PART 1, after question 4, if ‘yes’ on question 4. (Translated from Dutch to English).*
