# Supplementary material for: Safety and Comfort of an Innovative Drug Delivery Device in Healthy Subjects
Source: Transl Vis Sci Technol. 2020 Dec 18;9(13):35. doi: 10.1167/tvst.9.13.35 (PMC7757610; doi:10.1167/tvst.9.13.35)
Supplement: Supplement 6 [file tvst-9-13-35_s006.docx]

**Table S2.** Questionnaire during follow-up visits (30m, 8h, 24h, 48h, 7d, 14d, 21d, and 28d), translated from the Dutch version.

|  | Totally agree | Agree | Neutral | Disagree | Totally disagree | |
| --- | --- | --- | --- | --- | --- | --- |
| The ocular coil is not properly located in my eye. | 1 | 2 | 3 | 4 | 5 | |
| I feel the presence of the ocular coil in my eye. | 1 | 2 | 3 | 4 | 5 | |
| Presence of the ocular coil is uncomfortable | 1 | 2 | 3 | 4 | 5 | |
| Wearing the ocular coil made my vision blurry | 1 | 2 | 3 | 4 | 5 | |
| Wearing the ocular coil lowered my visual acuity | 1 | 2 | 3 | 4 | 5 | |
| Wearing the ocular coil made me close my eyes more often | 1 | 2 | 3 | 4 | 5 | |
| Wearing the ocular coil hinders me in during daily tasks | 1 | 2 | 3 | 4 | 5 | |
|  | Never | Sometimes | Often | Continuously | |  |
| My eye has a high tear production | 1 | 2 | 3 | 4 | |  |
| I have the intention to rub my eyes more often | 1 | 2 | 3 | 4 | |  |
| My eye itches | 1 | 2 | 3 | 4 | |  |
| My eye hurts | 1 | 2 | 3 | 4 | |  |
| I have eyestrain | 1 | 2 | 3 | 4 | |  |
| My eye feels irritated/It feels like there is sand in my eye. | 1 | 2 | 3 | 4 | |  |
| My eye feels burning | 1 | 2 | 3 | 4 | |  |

| Which grade would you give the ocular coil, according to the scaling on the right?  ……………………………………. |  Excellent  (not noticeable)  Highly comfortable  (sometimes noticeable)  Comfortable  (noticeable, not annoying)  Slightly uncomfortable  (noticeable and annoying)  Highly uncomfortable  (noticeable and severely annoying/ burning/ irritating)  Painful |
| --- | --- |
| Are there any other comments or remarks?  …………………………………….  …………………………………….  …………………………………….  …………………………………….  …………………………………….  …………………………………….  ……………………………………. |  |
